# Association between cognitive diagnosis and a range of significant life events in an elderly essential tremor cohort: a longitudinal, prospective analysis

**DOI:** 10.3389/fneur.2023.1193220

**Published:** 2023-06-14

**Authors:** Diane S. Berry, Diep Nguyen, Stephanie Cosentino, Elan D. Louis

**Affiliations:** ^1^Department of Neurology, University of Texas Southwestern Medical Center, Dallas, TX, United States; ^2^Taub Institute for Research on Alzheimer's Disease and the Aging Brain, Vagelos College of Physicians and Surgeons, Columbia University, New York, NY, United States; ^3^Department of Neurology, Vagelos College of Physicians and Surgeons, Columbia University, New York, NY, United States

**Keywords:** essential tremor (ET), dementia, mild cognitive impairment, significant life events, falls, hospitalizations

## Abstract

**Background:**

Although essential tremor (ET) patients have greater odds of mild cognitive impairment (MCI) and dementia than age-matched controls, the functional consequences of these enhanced odds are unknown. We examined associations between cognitive diagnosis and the occurrence of near falls, falls, use of a walking aid or a home health aide, non-independent living, or hospitalizations within a prospective, longitudinal study of ET patients.

**Methods:**

A total of 131 ET patients (mean baseline age = 76.4 ± 9.4 years) completed a battery of neuropsychological tests and questions about life events and were assigned diagnoses of normal cognition (NC), MCI, or dementia at the baseline and at 18-, 36-, and 54-month follow-ups. Kruskall–Wallis, chi-square, and Mantel–Haenszel tests assessed whether the diagnosis was associated with the occurrence of these life events.

**Results:**

Patients with final diagnoses of dementia were more often reported as living non-independently than NC or MCI patients and more often used walking aids than NC patients, with a *p-*value of <0.05. Patients with a final MCI or dementia diagnosis more often employed a home health aide than NC patients, with a *p*-value of <0.05. Moreover, Mantel–Haenzsel tests revealed linear associations between the occurrence of these outcomes and the level of cognitive impairment, with a *p*-value of <0.001 (i.e., dementia > MCI > NC).

**Conclusion:**

Cognitive diagnosis was associated with reported life events of ET patients, including the use of a mobility aid, employment of a home health aide, and removal from an independent living situation. These data provide rare insights into the important role cognitive decline plays in the experiences of ET patients.

## Introduction

Although the primary clinical feature in essential tremor (ET) is kinetic tremor (1), cognitive impairments have been associated with this disease (2–4). For example, studies have demonstrated that people with ET receive lower scores on standard cognitive tests than same-age healthy controls (5–7). Studies further documented that people diagnosed with ET are at greater risk of developing dementia than their same-age peers (8–10). Moreover, a substantial portion of the elder ET population is affected; a quarter (25.0%) have been identified to have prevalent dementia, (8) and more than a fifth (20.3%) have been identified to have mild cognitive impairment (MCI) (11).

Essential tremor is further associated with a variety of features that could have a functional impact. For example, people diagnosed with ET more commonly exhibit sleep disorders, including excessive daytime somnolence than controls (7, 12). They also can encounter more difficulties performing daily activities that are cognitively based (13–15) and often experience balance problems that increase their susceptibility to falls and, potentially, physical injury (16–18). Although these data suggest that ET might also be associated with a succession of additional significant life events, such as the requirement of a walking aid, the use of a home health aide, or moving to supervised living facilities, there is little research in this area (3).

We have a particular interest in the association between cognitive dysfunction in ET and such significant life events. For example, there is literature linking cognitive impairment in ET to fall risk (19–21). ET patients with dementia report more falls than non-dementia patients (21). However, additional links between cognitive performance and the significant life events experienced by ET patients have not been investigated (3, 15).

We present data from a prospective, longitudinal study of an ET cohort that examines the association between the cognitive diagnoses assigned to ET patients—normal, mild cognitive impairment, and dementia—and the experience of such significant life events, including near falls, falls, the use of a mobility aid, such as a wheelchair or walker, the use of a home health aide, living non-independently, and being admitted to the hospital.

## Methods

### Overview

Patients were enrolled in a prospective, longitudinal study of cognitive performance (Clinical Pathological Study of Cognitive Impairment in Essential Tremor, NINDS R01 NS086736) (21, 22). Eligibility requirements include (1) a diagnosis of ET; (2) a baseline age of at least 55 years; and (3) no history of brain surgery as a treatment for ET. Nationwide enrollment in COGNET began in July 2014, with patients being geographically widespread in more than 40 US states. Patients took part in an initial baseline evaluation, as well as in a second, third, and fourth (final) evaluation at the baseline plus 18, 36, and 54 months, respectively. The study was approved by Yale University, Columbia University, and the University of Texas Southwestern Medical Center Institutional Review Boards. All patients provided written, informed consent.

A trained research assistant administered the evaluations during home visits. Each involved the completion of demographic/clinical questionnaires, a battery of neuropsychological tests, and a videotaped neurological examination. Based on the videotaped examinations, an experienced movement disorders neurologist (E.D.L.) provided clinical diagnoses of ET derived from well-established reliable (23) and valid (24) criteria (25), as well as ratings of postural and kinetic tremor that provided the basis for calculating a Total Tremor Score (TTS). The TTS incorporated 12 individual ratings, each made on a 0–3 scale, yielding values that could range from 0 (low) to 36 (high).

### Neuropsychological test battery

During each study evaluation, patients completed a comprehensive battery of neuropsychological tests measuring performance in five broad cognitive domains. These required little or no reliance on motor functioning, minimizing any disadvantage to patients with moderate to severe tremors. The following tests were administered for each domain: (1) *Attention*, measured *via* the Wechsler Adult Intelligence Scale IV (WAIS-IV) Digit Span Forward (26), and the Oral Symbol-Digit Modalities Test (27); (2) *Executive function*, measured by the D-KEFS Sorting Test, the D-KEFS Verbal Fluency test, the D-KEFS Color Word Interference test, the D-KEFS Twenty Questions Test (28), and the Wechsler Adult Intelligence Scale IV (WAIS-IV) Digit Span Backward test (26); (3) *Language*, evaluated by scores on the Boston Naming Test (29); (4) *Memory*, assessed by the California Verbal Learning Test II (30), the Wechsler Memory Scale Revised: Logical Memory (31), and the Wechsler Memory Scale IV: Verbal Paired Associates Test (32); and (5) *Visuospatial*, assessed by the Benton Judgement of Line Orientation (33), the Benton Facial Recognition Test (34), and the WAIS IV Visual Puzzles test (26). For diagnostic purposes, raw scores were converted to z-scores adjusted based on clinically available normative data. Impairment on a given test was defined as a z-score of ≤ −1.5. In addition, specific tests in each domain were *a priori* selected for consideration in the subsequent diagnosis of MCI based on the following: (1) relative purity of measurement for the construct under evaluation; (2) the utility of measures for defining MCI in previous studies; and (3) general availability of the measures to researchers interested in replication (22).

Finally, patients completed the Montreal Cognitive Assessment (MOCA) (35) and the Mini-Mental State Exam (36), which are the measures of global cognitive performance.

### Independent variables: cognitive diagnosis

Following established protocols (21), patients were reviewed as part of a diagnostic consensus conference in which a neuropsychologist (S.C.) and a geriatric psychiatrist reviewed the comprehensive results of the study evaluation for each patient, including neuropsychological test performance, and subsequently assigned a Clinical Dementia Rating (CDR) score (0 = no dementia, 0.5 = questionable dementia, 1 = mild dementia, 2 = moderate dementia, and 3 = severe dementia) (37).

At each interval, patients were assigned diagnoses of either normal cognition (NC), MCI, or dementia (D). Normal cognition included (1) patients identified to have no impairment (CDR 0, no impairment on any test); (2) patients with impairment of unlikely clinical significance (CDR 0, impairment on 1 test); (3) patients with impairment of possible clinical significance (CDR 0 or 0.5, impairment in ≥1 test but not meeting operational criteria for MCI); and (4) patients with questionable or isolated functional impairment (CDR 0.5, no impairment on any test). MCI was operationally defined as a CDR of 0.5 and impairment (z-score ≤ −1.5) on two MCI-designated tests. Dementia was defined as a CDR of ≥1 and impairment in multiple domains.

Dementia diagnoses increased from 5 (3.8%) to 17 (13.0%) across the duration of the study (Table 1). Both the baseline and final evaluation cognitive diagnostic classifications served as independent variables; the final evaluation cognitive diagnostic classification essentially provided a cumulative measure of cognitive diagnosis, reflecting the diagnosis observed for each patient at the end of the study.

**Table 1:** Distribution of cognitive diagnosis at baseline and follow-up evaluations.

**Observation**	***N***	**Diagnosis**		
		**Normal cognition**	**Mild cognitive impairment**	**Dementia**
Baseline	131	109 (83.2)	17 (13.0)	5 (3.8)
Baseline plus 18-months	131	107 (81.7)	18 (13.7)	6 (4.6)
Baseline plus 36-months	131	106 (80.9)	15 (11.5)	10 (7.6)
Baseline plus 54 months	131	104 (79.4)	10 (7.6)	17 (13.0)
Net change from baseline to final evaluation	–	−5 (3.8)	−7 (5.4)	+12 (9.2)

Values = n (percentage). Row percentages equal 100%. *N* = 131.

### Outcome variables: life events

Patients reported (a) near falls, defined as the number of times during the past year that they felt they were going to fall, but did not really do so (data collected at evaluations 1, 2, 3, and 4); (b) falls, defined as the number of times they actually did fall during the past year (evaluations 1, 2, 3, and 4); (c) whether they were currently in a non-independent living arrangement (evaluations 1, 2, 3, and 4); (d) the number of times they were admitted to the hospital during the past 18 months (evaluations 2, 3, and 4); (e) whether or not they currently used a walking aid (i.e., a cane, walker, or wheelchair; evaluations 3 and 4); and (f) whether they engaged the services of a home health aide (evaluation 4). As seen above, as the study progressed, we expanded the protocol with several additional items to enrich our assessment of significant life events.

We created a cumulative version of each outcome variable, aggregating across evaluations to enhance the range and number of documented events. For example, the cumulative number of hospitalizations corresponds to the sum of the number of hospital admissions reported at evaluations 2, 3, and 4; the cumulative use of a walking aid indicates whether a patient reported its use at either evaluation 3 or 4.

### Study sample

Our sample consisted of 138 patients. Each received a clinical diagnosis of ET at the baseline and took part in all four observations. We excluded two patients who did not complete the cognitive battery, and five diagnosed with cognitive impairment due to injury, stroke, or substance abuse, yielding a final sample of 131 (61.1% women, mean age at baseline = 76.4 ± 9.4 years, and the mean age of tremor onset = 39.1 ± 21.1 years). The average time elapsed between baseline and follow-up (final) evaluation was 4.7 years.

### Statistical analyses

The distributions of cognitive diagnoses and demographic and clinical variables are shown in Tables 1, 2. Kruskall–Wallis, one-way analyses of variance, and chi-square tests were used to compare patients assigned to the three baseline cognitive diagnostic classifications (Table 2).

**Table 2:** Baseline cognitive diagnosis and demographic and clinical characteristics.

	**Total sample**	**Baseline diagnosis**			***p***
		**Normal cognition^1^**	**Mild cognitive impairment^1^**	**Dementia^1^**	
Age^1^ (Years)	76.4 ± 9.4	75.3 ± 9.4_a_	80.9 ± 7.9_b_	85.4 ± 4.3_b_	**0.004** ^**5**^
Sex (Female)	80 (61.1)	66 (60.0)	12 (70.6)	2 (40.0)	0.45^4^
Education (Years)	15.9 ± 2.4	16.0 ± 2.4	15.1 ± 2.6	15.6 ± 2.2	0.33^5^
Age tremor onset (Years)	39.1 ± 21.1	39.1 ± 20.8	40.4 ± 21.8	33.3 ± 30.4	0.80^5^
Tremor duration^2^ (Years)	37.1 ± 20.9	36.0 ± 20.4	40.5 ± 20.5	51.3 ± 33.8	0.38^5^
TTS^3^	23.8 ± 5.4	23.5 ± 5.4	24.3 ± 5.2	27.6 ± 4.0	0.21^5^
MMSE	28.8 ± 1.6	29.1 ± 1.4_a_	28.2 ± 1.6_b_	25.0 ± 2.5 _c_	**0.001** ^**5**^
MOCA	25.2 ± 3.1	25.9 ± 2.6_a_	22.2 ± 2.7_b_	19.0 ± 3.2 _c_	**0.001** ^**6**^

*N* = 131. Baseline N normal cognition = 109, mild cognitive impairment = 17, and dementia = 5. Values represent mean ± standard deviation or n (percentage). TTS, total tremor score; MMSE, Mini-Mental State Exam; MOCA, Montreal Cognitive Assessment. Given a significant overall *p-*value, subscripts identify which specific pairs of values within a given row are significantly different. Values that share a common subscript do not differ at a *p*-value of <0.05. Conversely, values that do not share a common subscript differ at a *p*-value of <0.05. ^1^Baseline assessment. ^2^Tremor duration = Baseline age minus the age of tremor onset. ^3^TTS baseline assessment ranges from 0 (low) to 36 (high). ^4^Chi-square test; bolded values are significant at *p* < 0.05. ^5^Kruskall–Wallis test; bolded values are significant at *p* < 0.05. ^6^Analyses of variance; bolded values are significant at *p* < 0.05.

The Kruskall–Wallis test compared the cumulative frequency with which patients assigned to each cognitive diagnosis at baseline reported falls, hospitalizations, and near falls (Table 3). The chi-square test compared the proportion of patients assigned to each baseline cognitive diagnosis of those who reported vs. those who did not report the employment of a home health professional, participation in a non-independent living arrangement, or use of a walking aid. The Mantel–Haenszel test of trend in which the three baseline diagnosis groups (normal cognition, MCI, and dementia) were treated as an ordinal scale of cognitive impairment additionally tested for the presence of linear associations between diagnosis and the occurrence of the dichotomous events (Table 3).

**Table 3:** Baseline cognitive diagnosis and cumulative measures of significant life events.

	**Baseline diagnosis**			**Statistic**	***p*-values**
	**Normal cognition**	**Mild cognitive impairment**	**Dementia**		
**Continuous outcome variables**					
Falls	2	4	1	2.66^1^	0.26
Hospitalizations	1	1	0	0.41^1^	0.81
Near falls	19.8	57	n/a^4^	0.06^1^	0.8
**Dichotomous outcome variables**					
Home health aide (Yes)	11 (10.4)	3 (18.8)	1 (33.3)	2.25^2^	0.33
				2.17^3^	0.14
Non-independent living (Yes)	20 (18.3)_a_	8 (47.1)_b_	2 (40.0)_ab_	7.72^2^	**0.02**
				6.05^3^	**0.01**
Walking aid (Yes)	15 (13.8)_a_	5 (29.4)_a_	5 (100.0)_b_	24.37^2^	**0.001**
				19.82^3^	**0.001**

Values represent the median for continuous variables and N (percentage) for dichotomous variables. N's for normal cognition, mild cognitive impairment, and dementia groups at baseline equal 109, 17, and 5, respectively. DF for particular outcome measures may differ slightly due to missing data. Cumulative continuous outcome measures equal to the total n of a given event collapsing across all evaluation periods. For cumulative dichotomous outcome measures, 1 = event did occur during the duration of the study, 0 = event did not occur during the duration of the study. Given a significant overall *p-*value, subscripts identify which specific pairs of values within a given row are significantly different. Values that share a common subscript do not differ at a *p*-value of <0.05. Conversely, values that do not share a common subscript differ at a *p*-value of <0.05. ^1^Kruskall–Wallis test, H statistic; bolded *p*-values are significant at *p* ≤ 0.05. ^2^Chi-square test, *X*^2^ statistic; bolded *p*-values are significant at *p* ≤ 0.05. ^3^Mantel–Haenszel test of trend, *df* = 1; bolded *p*-values are significant at *p* ≤ 0.05. ^4^No data on near falls available for patients assigned to this group.

In a parallel set of analyses, Kruskall–Wallis, chi-square, and Mantel–Haenszel tests further examined relations between cognitive diagnoses assigned at the final observation and the cumulative continuous and dichotomous outcome variables (Table 4).

**Table 4:** Final cognitive diagnosis and cumulative measures of significant life events.

	**Final diagnosis**			**Statistic**	***p-values***
	**Normal cognition**	**Mild cognitive impairment**	**Dementia**		
**Continuous outcome variables**					
Falls	2	3.5	2	1.16^1^	0.56
Hospitalizations	1	1	0.5	0.34^1^	0.85
Near falls	21.5	24	0	1.98^1^	0.37
**Dichotomous outcome variables**					
Home health aide (Yes)	7 (6.9)_a_	3 (30.0)_b_	5 (35.7)_b_	12.98^2^	**0.002**
				12.29^3^	**0.001**
Non-independent living (Yes)	17 (16.3)_a_	2 (20.0)_a_	11 (64.7)_b_	19.41^2^	**0.001**
				17.24^3^	**0.001**
Walking aid (Yes)	11 (10.6)_a_	3 (30.0)_ab_	11 (64.7)_b_	28.56^2^	**0.001**
				28.02^3^	**0.001**

Values represent medians for continuous variables and N (percentage) for dichotomous variables. N's for normal cognition, mild cognitive impairment, and dementia groups at final evaluation equal 104, 10, and 17, respectively. DF for particular outcome measures may differ slightly due to missing data. Cumulative continuous outcome measures equal to the total n of a given event collapsing across all evaluation periods. For cumulative dichotomous outcome measures, 1 = event did occur during the duration of the study and 0 = event did not occur during the duration of the study. Given a significant overall *p-*value, subscripts identify which specific pairs of values within a given row are significantly different. Values that share a common subscript do not differ at a *p-*value of <0.05. Conversely, values that do not share a common subscript differ at a *p*-value of <0.05. ^1^Kruskall–Wallis test, H statistic, *df* = 2; bolded *p*-values are significant at a *p*-value of ≤ 0.05. ^2^Chi-square test, *X*^2^ statistic, *df* = 2; bolded *p*-values are significant at a *p-*value of ≤ 0.05. ^3^Mantel–Haenszel test of trend, *df* = 1; bolded *p*-values are significant at a *p*-value of ≤ 0.05.

Finally, a series of sensitivity analyses were conducted to assess the impact of various patient characteristics on the outcome measures. In each, a subset of patients was excluded from analyses based on their characteristics. An identical set of analyses to those just described was then conducted on the remaining patients to assess whether the pattern of effects differed when a given subset of patients was excluded.

## Results

### Cognitive diagnoses and demographic and clinical measures

The distribution of cognitive diagnoses is shown at the baseline and at each follow-up evaluation (Table 1). Patients assigned to NC, MCI, and dementia diagnoses at the baseline did not vary in sex, years of education, age of tremor onset, tremor duration, or tremor severity score (TTS), with a *p*-value of ≥ 0.21. Baseline NC patients were younger than either MCI or dementia patients, with a *p*-value of <0.05 (Table 2). NC patients also received higher MMSE and MOCA scores at the baseline than patients diagnosed with MCI, with a *p*-value of ≤ 0.05, and MCI patients received higher baseline MMSE and MOCA scores than patients with a diagnosis of dementia, with a *p*-value of ≤ 0.05 (Table 2).

### Baseline cognitive diagnosis and cumulative significant life events

Neither the cumulative number of falls, hospital admissions, or near falls nor the proportion of patients employing a home health aide during the study was associated with baseline cognitive diagnostic classification, with a *p*-value of ≥ 0.26 (Table 3). However, the total proportion of patients who participated in a non-independent living arrangement and used a walking aid did differ as a function of baseline cognitive diagnosis, with a *p*-value of ≤ 0.02 (Table 3). More specifically, a lower percentage of NC patients reported non-independent living arrangements (18.3%) than MCI patients (47.1%), with a *p*-value of <0.05. In addition, the percentage of dementia patients using a walking aid (100.0%) was significantly greater than the percentage of either MCI (29.4%) or NC (13.8%) patients doing so, with a *p*-value of ≤ 0.05.

The Mantel–Haenszel test of trend revealed parallel results for the cumulative dichotomous outcome variables. More specifically, significant linear associations were observed between increasing levels of cognitive impairment at the baseline (i.e., normal cognition, MCI, dementia) and the occurrence of non-independent living arrangements, with a *p*-value of ≤ 0.01, and of walking aid use, with a *p*-value of ≤ 0.001. These tests revealed no significant association between the use of a home health aide and baseline level of cognitive impairment, with a *p*-value of ≥ 0.14 (Table 3).

### Final cognitive diagnosis and cumulative significant life events

The cumulative number of falls, hospital admissions, and near falls did not differ as a function of the final diagnosis, with a *p*-value of ≥ 0.37 (Table 4). However, the total proportion of patients who employed a home health professional, who participated in a non-independent living arrangement, or who used a walking aid did differ as a function of diagnosis, with a *p*-value of ≤ 0.002 (Table 4). Specifically, the percentage of NC patients employing a home health aide (6.9%) was significantly lower than the percentage observed for either MCI (30.0%) or dementia patients (35.7%), with a *p*-value of ≤ 0.05. The percentage of NC and MCI patients reporting a non-independent living arrangement (16.3 and 20.0%, respectively) was significantly lower than the percentage of dementia patients reporting such an arrangement (64.7%), with a *p*-value of ≤ 0.05. Finally, a lower percentage of NC patients than dementia patients reported using a walking aid, 10.6 and 64.7%, respectively, with a *p*-value of <0.05.

The Mantel–Haenszel test revealed parallel results for the cumulative dichotomous outcome variables. More specifically, linear associations were observed between increasing levels of cognitive impairment at the final evaluation (i.e., normal cognition, MCI, and dementia) and use of a home health aide, with a *p*-value of ≤ 0.001, engagement in non-independent living arrangements, with a *p*-value of ≤ 0.001, and the use of a walking aid, with a *p*-value of ≤ 0.001 (Table 4).

### Sensitivity analyses

We conducted follow-up analyses to examine potential moderators of the relations between cognitive diagnosis and our outcome measures. First, we considered the possibility that the use of rivastigmine, an acetylcholinesterase inhibitor, used to treat dementia and documented to improve gait (38), might influence the relationship between the level of cognitive impairment and life events. Only one dementia patient was reported using rivastigmine. We conducted parallel sets of analyses to those described previously, testing associations between baseline diagnoses and outcome variables, and between final diagnoses and outcome variables; these analyses excluded the patient who reported the use of rivastigmine. The results of these analyses were nearly identical to those previously described.

Second, as up to a third of individuals with MCI may revert to normal cognition within a year (39, 40), we considered whether patients characterized by such instability might influence our results. We identified four individuals who reverted to NC subsequent to a diagnosis of MCI. We conducted parallel sets of analyses to those described previously excluding data from these four patients. The results of these analyses were nearly identical to those previously described.

Finally, we considered the possible effects of conversion to Parkinson's disease (PD) on our results. Specifically, we identified three patients in our sample who developed a diagnosis of ET/PD on the final observation. Again, we carried out parallel sets of analyses excluding data from these three patients, and the results of these analyses were nearly identical to those previously described.

## Discussion

The focus of this longitudinal, prospective study was the identification of links between cognitive diagnosis (normal cognition, MCI, or dementia) and the experience of significant life events during the course of the study. Patients with a diagnosis of dementia at baseline and final evaluation were more likely to use a walking aid than their NC peers. Baseline MCI patients and dementia patients at final evaluation were more likely than their NC peers to live in a non-independent arrangement. Finally, patients with a final MCI or dementia diagnosis were more likely than others to employ a home health aide during the study. No relations were observed between either baseline or final diagnosis and the occurrence of near falls, falls, or hospital admissions. In addition, the Mantel–Haenszel test of trend revealed a significant linear association between an increase in cognitive impairment (i.e., in classifications of NC, MCI, and dementia, respectively) and the occurrence of non-independent living and the use of a walking aid at the baseline and the occurrence of non-independent living and the use of a walking aid, and the use of a home health aide at final evaluation.

Associations between cognitive impairment and certain clinical outcomes (e.g., hospital admission, care home admissions) have been reported in patients with Parkinson's or Alzheimer's disease (3, 41–44). However, to the best of our knowledge, these are the only available data both documenting and quantifying the nature of parallel links in patients with ET (3). Moreover, our data suggest that some of these associations, at least in ET patients, may develop even at pre-dementia levels of cognitive impairment, that is, among ET patients diagnosed with MCI.

Thus, ET patients experiencing sufficient levels of cognitive impairment to render a diagnosis of MCI or dementia may also require higher than normal levels of support in the performance of their daily activities (e.g., non-independent living). Whereas the tremor that accompanies ET typically detracts from the performance of routine daily tasks *via* motor skill disruption, ET-related cognitive decline instead undermines the performance of daily activities *via* the disruption of the ability to plan, recall and successfully execute goal directed behaviors. Understanding the progressive nature of these disruptions may help patients and their families anticipate and better prepare for such events.

The lack of association between cognitive diagnosis and hospital admissions seems counterintuitive, and the lack of relationship between diagnosis and measures of falls and near falls is not consistent with previous work (20, 21). Although there was reasonable variability in the number of near falls reported during the study (sample median = 21.5), falls and hospitalizations are relatively rare events, and their low numbers may make the identification of relations between these two variables and diagnosis difficult. In fact, this particular concern led us to use the *cumulative* numbers of events that occurred throughout the entire study period as outcome measures. Although this approach increased the range and average frequency of these events, a low number of each event was still typically observed (sample median = 1.0 and 2.0 for hospitalizations and falls, respectively).

The lower than expected rates of near falls, falls, and hospitalizations among our patients with either MCI or dementia may further result, in part, from the greater use of protective measures that we document among them, i.e., the greater popularity of walking aids, such as wheelchairs, canes, and walkers, more frequent engagement of the services of home health professionals, and the more common use of non-independent living arrangements. For example, although we know that ET patients diagnosed with dementia have greater balance problems than their peers, (21) dementia patients in our sample were also more likely than cognitively normal patients to use a walking aid while navigating a more highly supervised and supported environment. This may have reduced the likelihood that any existing problems with balance would directly translate into either near falls or falls. Not unlike other brain donor samples (45, 46), the cohort from which our dementia patients were drawn is highly educated (*M* = 15.86 years) and hails from a population particularly likely to seek medical assistance. Thus, we caution that other samples of ET patients diagnosed with dementia may be less likely to employ such protective measures, and, as a result, (unfortunately) display comparatively greater numbers of falls or hospitalizations. Additional research should address this possibility as well as study other populations with different and more heterogeneous features. Finally, as patients in our sample were willing and able to participate in four evaluations over a period of nearly 5 years, they may have been healthier and more socially engaged than a random sample of ET patients.

These data represent the only longitudinal assessment of the relations between cognitive functioning and these significant life events in an ET cohort. An additional strength of this study is that patients were selected for inclusion based on ET diagnoses carefully assigned by a senior movement disorders neurologist after reviewing a detailed neurological examination. In addition, cognitive diagnoses were based on comprehensive assessments that were thoroughly evaluated by both a neuropsychologist and a geriatric psychiatrist. Moreover, these cognitive assessments required little or no reliance on motor skills, ensuring that patients with substantial tremors were not placed at a disadvantage.

These analyses explore the spectrum of effects associated with cognitive diagnoses (MCI and dementia) in patients with ET. Our goal is to provide guidance to these patients regarding the likelihood that they will experience such life events. Hence, our present focus is on the expression of cognitive impairment within an ET population. Inherent in these analyses is the notion that cognitive impairment in ET is not self-limited, isolated, and benign, but rather it is associated with a potential host of other consequences. A somewhat different but important goal of future research should be the comparison of that expression in an ET vs. a non-ET population. This will require the longitudinal study of these questions within a design that includes both ET patients and control participants.

Our data suggest that ET patients with dementia may be especially likely to live in an environment designed to counterbalance their increased frailties—and, in turn, their experience of certain significant events. Moreover, this study provides insight into the important but largely unexplored correlates and consequences of cognitive decline in an ET cohort. We hope that clinicians can use these data to help patients and their families anticipate the challenges to daily living associated with ET.

## Data availability statement

The raw data supporting the conclusions of this article will be made available by the authors, without undue reservation.

## Ethics statement

The studies involving human participants were reviewed and approved by Yale University, Columbia University, and University of Texas Southwestern Medical Center Institutional Review Boards. The patients/participants provided their written informed consent to participate in this study.

## Author contributions

DB and EL contributed to the conception and design of the study and manuscript revision. DN organized the data base. SC designed the cognitive assessments. DB performed the statistical analyses and wrote the first draft of the manuscript. All authors have read and approved the submitted versions.
